# Healthcare Resource Management and Pandemic Preparedness for COVID-19: A Single Centre Experience From Jodhpur, India

**DOI:** 10.34172/ijhpm.2020.102

**Published:** 2020-06-20

**Authors:** Satyendra Khichar, Naresh Midha, Gopal Krishana Bohra, Deepak Kumar, Maya Gopalakrishanan, Bharat Kumar, Varatharajan Sakthivadivel, Mahendra Kumar Garg

**Affiliations:** All India Institute of Medical Sciences, Jodhpur, India.

## Dear Editor,

Coronavirus disease 2019 (COVID-19) pandemic is overwhelming health resources, leading to critical shortages of healthcare workers (HCWs), space and supplies with serious implications for patient outcome.^1^ Developing countries with overburdened healthcare systems face a greater challenge.^2^ Hence, judicious utilization of healthcare resources and strategic planning are important to prevent spread of infection and maintain health-system resilience.^3^

Most countries have come up with national level pandemic guidance which are being adapted by hospitals for their own preparedness. The first case of COVID-19 in India was reported on 30 January and the Indian Government imposed International travel restrictions and nation-wide Lockdown by third week of March 2020. The key focus, in the several reports from India, South Korea and Nepal, are on health-resource allocation and infection prevention. Here we describe the various aspects of pandemic preparedness undertaken at our centre an 800 bedded tertiary-care hospital at Rajasthan, India catering to a population of 12 million. Daily flow is approximately 1500 patients at outpatient and 200 at emergency with facilities for COVID-19 testing. We share our experiences during the pandemic with focus on workflow and human resource management.

With proactive preventive measures, we expect about 20 000 patients over 4 months, of which 400 may require critical care in a month including 100, which will be managed by us.^4^

Focus areas for preparedness were (*a*) Healthcare-space management, (*b*) Healthcare personnel management, (*c*) Training of HCWs and (*d*) Infection prevention in HCWs. The institute formed a COVID-19 pandemic preparedness core-committee comprising of representatives from all departments and the administration to plan and execute activities of patient-care, infection-prevention, pharmacy, laboratory, security, engineering, maintenance, waste-management. A nodal officer was nominated to liaise with government administrative authorities.

**Figure 1:**
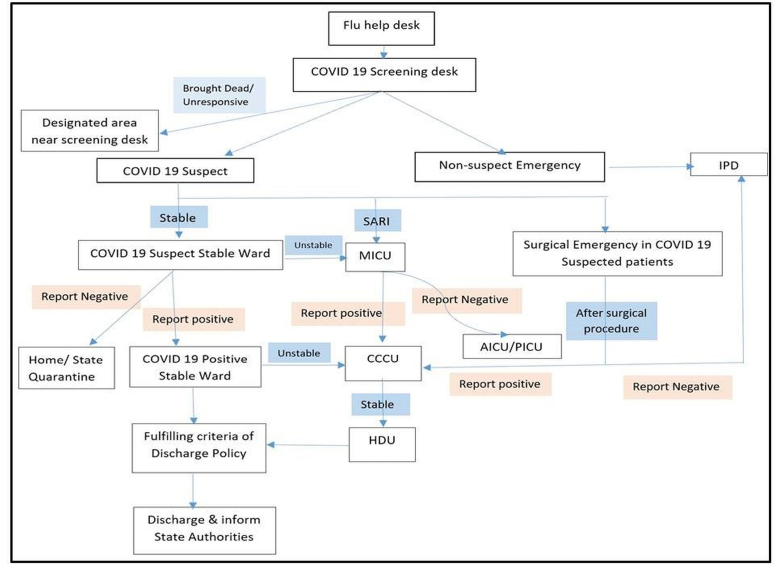
Flowchart Showing Workflow Management. Abbreviations: IPD, inpatient department; SARI, severe acute respiratory infection; MICU, medicine intensive care unit; AICU, adult intensive care unit; PICU, paediatric intensive care unit; CCCU, COVID-19 critical care unit; HDU, COVID-19 high dependency unit.

The hospital was divided into 5 zones: COVID-19 Screening desk (Triage zone), Wards for COVID-19 suspected patients, Wards for COVID-19 stable patients, COVID-19 Critical care units (CCCUs) and Emergency services for non-COVID-19 patients (Figure 1). All COVID-19 designated areas had specific rooms for donning and doffing of personal protective equipment (PPE). We suspended elective surgeries and routine outpatient services. Beds allocated for COVID-19 were 410 including 100 critical care beds.

**Figure 2:**
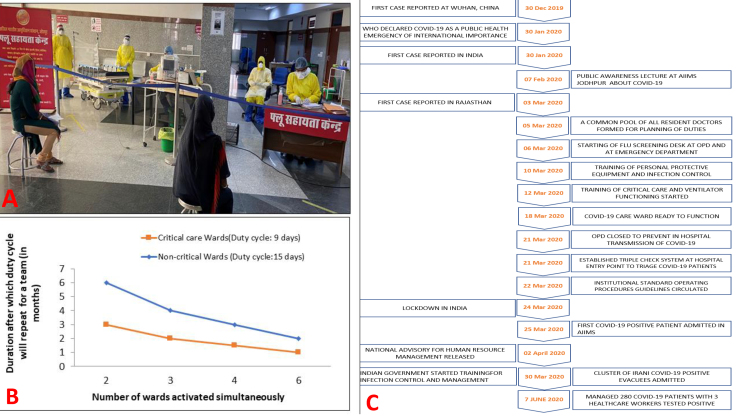
(A) Photograph showing COVID-19 screening desk with health workers in PPE, transfer trolley and yellow line marked on floor. (B) Graph depicting surge management plan (C) Timeline comparing COVID-19 world events with Institutional response. Abbreviations: COVID-19, coronavirus disease 2019; PPE, personal protective equipment.

Workflows were managed by a single-point hospital entry.^5^ A triple check system with: flu-help desk, COVID-19 screening desk followed by emergency physician evaluation was arranged. Patients were given surgical masks and educated about preventive measures. Separate areas for COVID-19 suspected patients requiring surgical or obstetrics care were designated. Pathways from screening desk to COVID-19 zones were marked with yellow lines and a separate lift was assigned for transportation of patients. A hospital attendant with designated trolley in PPE transferred patients (Figure 2).

Deployment of residents and nursing personnel was planned in groups according to core speciality and capability at various working zones.^6^ We describe the plans for resident doctor deployment. Similar plans were made for other HCWs. Each day was divided in three shifts of 8 hours. Those working in COVID-19 zones are provided with separate facilities for stay and food at hospital campus during duty days to avoid mingling with other HCWs.

We constituted teams comprising of junior residents (postgraduate trainees) and senior residents (Post M.D.) according to working zones (Table). For example, residents from clinical departments with critical-care experience are posted in CCCU while non-clinical residents are posted at screening desk and stable COVID-19 zones (Table). A 9 days duty-cycle was assigned to residents in CCCU and 15 days duty cycle in other COVID-19 wards followed by 14 days quarantine. Surge plans were prepared for activating multiple wards/teams simultaneously in case of exponential patient admission.^7^ These plans were communicated in advance to the duty residents and their departments to allow flexibility (Figure 2). Teams were serially activated as and when patients were admitted to the COVID-wards.

**Table:** Deployment of Resident Doctors with Supportive Staff and Their Working Zones

**Groups**	**Core Speciality of Duty Doctors**	**Working Zone/Numbers of Beds** ^c^	**Doctors**	**Teams of Duty Doctors** ^e^	**Manpower Per Shift of 8 Hours** ^e^ **(Each Ward)**
Group A	Non-Academic Junior Resident (MBBS) doctors	COVID-19 screening desk	32	8	Duty doctor – 1 Nursing officer – 1 Hospital attendant – 1 House keeping staff – 1 Data entry operator – 1
Group B^a^	Resident doctors of clinical and para clinical specialties not dealing with Intensive care	COVID-19 suspected ward (4 wards/total 80 beds) COVID-19 positive stable patients ward (6 wards/total 120 beds)	100	25	Duty doctor – 1 (JR) Nursing officer – 1 Hospital attendant – 1 House keeping staff – 1
Group C^b^	Senior and junior resident doctors of medical and surgical specialties already giving intensive care	CCCU^d^ (4 wards/100 beds including step down beds)	80 Senior resident doctors and 80 junior resident doctors	20	Duty doctor – 2 (1 SR + 1 JR) Senior nursing officer – 1 Nursing staff – 1 per 2 beds Hospital attendants – 2 House keeping staff – 2

Abbreviations: MBBS, Bachelor of Medicine and Bachelor of surgery; JR, Junior Resident (Postgraduate trainee); SR, Senior Resident (Post MD/MS); CCCU: COVID-19 critical care unit; COVID-19, coronavirus disease 2019. ^a^ Group B included resident doctors from the following specialities: Anatomy, Physiology, Biochemistry, Pharmacology, Pathology, Microbiology, Forensic Medicine, Eye, Otolaryngology, Psychiatry, and Gynaecology. ^b^ Group C – Resident doctors from Medicine, Surgery, Paediatrics, Anaesthesia, Cardiology, Cardiothoracic surgery, Neurology, Neurosurgery and Urology. ^c^100 beds in a block are prepared as reserve beds for COVID-19 surge. ^d^Ten beds are allotted for suspected COVID-19 patients in Medical ICU. ^e^For 8 hours shift, a team of 3 duty doctors with 1 reserve doctor per day are posted for 9 days in critical wards and 15 days for other wards.

All HCWs were trained in 3 modules - standard operating procedures with transfer protocols, infection control measures including PPE donning-doffing and endotracheal intubation/mechanical ventilator management. In these sessions, patient communication was also emphasized. A module for psychosocial wellbeing of HCWs was arranged.

Infection control measures included greeting with folded hands rather than handshakes, termed as “NAMASTE” campaign and social distancing. Donning/Doffing areas and patient movement paths were marked within COVID-19 zones. Negative pressure for COVID-19 wards was created on temporary basis. Teleconferencing facilities were installed for communication with stable patients to reduce HCW exposure time. Reusable PPEs designed in collaboration with Indian Institute of Technology, Jodhpur, ensured sufficient availability.^8^

As on June 7, 2020, we have managed 856 COVID-19 suspect, 280 COVID-19 positive patients with two deaths. All HCWs including 452 resident-doctors, 176 faculties and 900 nursing and allied staff have successfully completed the training. As on date, we have only three cases of infection in HCWs who were isolated early, preventing further spread. All contacts of these HCW were line-listed and tested, none of whom came positive implying that strict infection control and training measures are useful.

Strengths of our plan include the early actions we have initiated compared to national pandemic response as illustrated in Figure 2. However, still in the early days of pandemic, our experience so far suggests that careful planning maybe the key to weather the crisis.

## Acknowledgements

We thank Dr. Shashikant Saini, intern, Department of Internal Medicine for his help with the figure illustrations.

## Ethical issues

Not applicable.

## Competing interests

Authors declare that they have no competing interests.

## Authors’ contributions

SK, NM, DK, GKB wrote the first draft of the manuscript. MG, BK, and VS were involved in editing and revision of the draft. GKB and DK are in-charge of screening desk and triage. DK, SH, and MG are involved actively in providing clinical care to COVID-19 patients. MKG has provided overall guidance for clinical care as well as pandemic preparedness.
